# LiCl induces TNF-α and FasL production, thereby stimulating apoptosis in cancer cells

**DOI:** 10.1186/1478-811X-9-15

**Published:** 2011-05-24

**Authors:** Larissa Kaufmann, Gabriela Marinescu, Irina Nazarenko, Wilko Thiele, Carolin Oberle, Jonathan Sleeman, Christine Blattner

**Affiliations:** 1 Karlsruher Institute of Technology, Institute of Toxicology and Genetics, PO-Box 3640, 76021 Karlsruhe, Germany; 2 University of Heidelberg, Medical Faculty Mannheim, Ludolf-Krehl-Str. 13-17, 68167 Mannheim, Germany

## Abstract

**Background:**

The incidence of cancer in patients with neurological diseases, who have been treated with LiCl, is below average. LiCl is a well-established inhibitor of Glycogen synthase kinase-3, a kinase that controls several cellular processes, among which is the degradation of the tumour suppressor protein p53. We therefore wondered whether LiCl induces p53-dependent cell death in cancer cell lines and experimental tumours.

**Results:**

Here we show that LiCl induces apoptosis of tumour cells both *in vitro *and *in vivo*. Cell death was accompanied by cleavage of PARP and Caspases-3, -8 and -10. LiCl-induced cell death was not dependent on p53, but was augmented by its presence. Treatment of tumour cells with LiCl strongly increased TNF-α and FasL expression. Inhibition of TNF-α induction using siRNA or inhibition of FasL binding to its receptor by the Nok-1 antibody potently reduced LiCl-dependent cleavage of Caspase-3 and increased cell survival. Treatment of xenografted rats with LiCl strongly reduced tumour growth.

**Conclusions:**

Induction of cell death by LiCl supports the notion that GSK-3 may represent a promising target for cancer therapy. LiCl-induced cell death is largely independent of p53 and mediated by the release of TNF-α and FasL.

Key words: LiCl, TNF-α, FasL, apoptosis, GSK-3, FasL

## Background

Tumour necrosis factor alpha (TNF-α) is a cytokine that is mainly secreted by activated macrophages, although other cell types can also produce this protein in response to certain stimuli [1]. After binding to its corresponding transmembrane receptor, tumour necrosis factor receptor (TNF-R), TNF-α exerts cytostatic and cytotoxic activity against a wide range of human and murine cell lines [2,3]. Binding of TNF-α to its receptor induces receptor trimerisation on the cell surface and formation of a death-inducing signalling complex (DISC) at the cytoplasmic tail of TNF-R, leading to activation of Caspase-8 and induction of apoptosis [4]. Similar to TNF-α, FasL stimulates the formation of a DISC upon binding to its receptor (reviewed in: [5]) and induces cell death.

LiCl (lithium chloride), the lithium salt of hydrochloric acid is an important therapeutic agent for the treatment of patients suffering from bipolar disorder and depression [6]. Its main cellular target is Glycogen synthase kinase-3 (GSK-3). At least at physiological doses, LiCl has no effect on other protein kinases [7]. GSK-3 is a serine/threonine kinase that was initially identified as a regulator of glycogen synthase [8,9]. Mammals possess two isoforms of GSK-3 (α and β) [10]. Unlike most other protein kinases, GSK-3 is constitutively active in resting cells. Exposure to insulin, epidermal growth factor, ionizing radiation or phorbol ester, however, leads to rapid inactivation of GSK-3, which constitutes a determinant of embryonic development and cell fate [11-14]. Apart from LiCl, GSK-3 is efficiently inhibited by paullones, amongst which alsterpaullone is the most specific derivative [15].

GSK-3 phosphorylates several cellular substrates, including transcription factors such as c-Jun, c-Myb, CREB (cAMP response element binding protein) and Mdm2 [11,16-19]. Mdm2 is a ubiquitin ligase for the p53 tumour suppressor protein and some other targets [20]. GSK-3 phosphorylates the Mdm2 protein in its central domain and this phosphorylation is essential for Mdm2-mediated degradation of the p53 protein [11]. Accordingly, inhibition of GSK-3 leads to the accumulation of p53 and transcription of its target genes [11].

Since p53 is a protein with strong anti-proliferative and pro-apoptotic activities [21], we speculated that inhibition of GSK-3 may prevent cell proliferation and induce cell death in cells with wild type p53. Here we show that LiCl is a potent inducer of apoptosis both *in vitro *and *in vivo*. Although the presence of p53 slightly modifies the response, this tumour suppressor protein is not required for induction of cell death by LiCl. Moreover, we report that a major way in which LiCl induces apoptosis is by inducing autocrine production of TNF-α and FasL, thereby activating the extrinsic apoptotic pathway.

## Results

### LiCl and alsterpaullone prevent proliferation of tumour cells

Previous investigations showed that inhibition of GSK-3 leads to the accumulation and activation of p53 [11,22], a tumour suppressor protein that induces cell cycle arrest and apoptosis. With this in mind, we investigated the consequence of GSK-3 inhibition on the proliferation of tumour cells.

**Figure 1:**
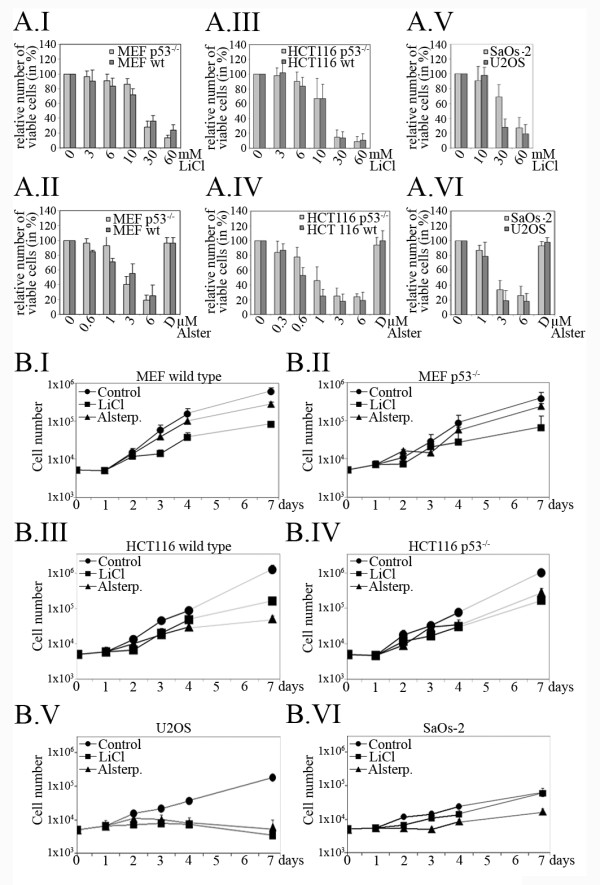
**Treatment of cells with LiCl and alsterpaullone reduces cell proliferation**. **(A.I-A.VI)** Wild type (wt) and p53-deficient mouse embryonic fibroblasts (MEF) and HCT116 cells as well as U2OS and SaOs-2 cells were plated in 96-well plates at a density of 1 × 10^3 ^cells per well. 24 hours after plating, LiCl and alsterpaullone (Alster) were added at the indicated concentrations. 72 hours after drug addition, the relative number of viable cells was determined by MTT-assay. Mean values and standard deviations of three independent experiments were calculated and plotted. The relative number of cells in the absence of drug was set to 100% (D: DMSO control). **(B.I-B.VI) **Wild type (wt) and p53-deficient mouse embryonic fibroblasts (MEF) and HCT116 cells as well as U2OS and SaOs2 cells were plated in 24-well plates at a density of 5 × 10^3 ^cells per well. 24 hours after plating, LiCl and alsterpaullone (Alster) were added at a concentration approximating LD_50 _as determined by the MTT assay (A.I-A.VI). U2OS cells and MEFs received a final concentration of 17.5 mM LiCl or 1.5 μM alsterpaullone, SaOs-2 cells were treated with 25 mM LiCl or with 1.5 μM alsterpaullone and HCT116 cells received 12.5 mM LiCl and 0.6 μM alsterpaullone. Cells were counted at the indicated days. Mean values and standard deviations of three independent experiments were calculated and plotted.

We incubated the human colon carcinoma cell line HCT116, and the two human osteosarcoma cell lines U2OS and SaOs-2 as well as mouse embryonic fibroblasts (MEFs) with increasing doses of LiCl and alsterpaullone and determined relative cell proliferation by MTT assay. Since we were particularly interested whether an eventual induction of cell death would require the p53 protein, we used HCT116 and MEF wild type cell lines and corresponding cell lines with a genetic deletion of p53. In addition, we employed the two osteosarcoma cell lines U2OS (p53 wild type) and SaOs-2 (p53-deficient) which differ in their p53 status. In Additional file 1, Figure S1, we show that p53 is only expressed in the wild type counterparts of HCT116 and MEF as well as in U2OS but not in the derivatives with deleted p53 alleles or in SaOS-2. As shown in Figure 1A I-VI, treatment of the different cell lines with LiCl strongly reduced cell proliferation in a dose dependent manner. Similar results were obtained with HaCaT, RKO and Hela tk- cell lines (data not shown). For MEF and HCT116 cells, we observed a decrease in the number of viable cells starting from about 3 mM LiCl. The half-lethal dose (LD_50_) for both cell lines was between 10 and 30 mM LiCl. SaOs-2 and U2OS cells required higher doses. Here, a clear decrease in the relative number of viable cells was only reached beyond 10 mM. The LD_50 _for U2OS was between 10 and 30 mM and for SaOs-2 between 30 and 60 mM. In all cases, we observed a slightly higher sensitivity of p53-replete cells than of p53-deficient cells, particularly at lower doses (Figure 1A I-VI). When we treated U2OS and HCT116 cells with the p53 inhibitor pifithrin-α [23], we also observed a slightly reduced sensitivity (Additional file 2, Figure S2A), signifying a contribution of p53 to LiCl-mediated cell death. However, when we knocked p53 down by siRNA, we found at best a slightly increased survival in the presence of p53 (Additional file 2, Figure S2B), supporting the notion that p53 is not an important mediator of LiCl-induced cell death.

When we investigated the colony forming ability of HCT116 cells, we observed a slightly higher sensitivity for LiCl than found with the MTT assay. Here, proliferation of p53-replete cells was already slightly reduced at 1 mM LiCl, while p53-deficient cells required at least 3 mM LiCl for inhibition of colony forming ability (Additional file 3, Figure S2C).

To investigate whether the reduction in proliferation in response to LiCl might be due to inhibition of GSK-3, we repeated these experiments with another inhibitor of GSK-3, alsterpaullone. Here, proliferation of HCT116 was already reduced at a dose of 0.3 μM alsterpaullone while the other cell lines required at least 1 μM of the drug for growth suppression (Figure 1A I-VI). LD_50_s were at about 3 μM for MEFs, between 0.6 and 1 μM for HCT116 and between 1 and 3 μM for the two osteosarcoma cell lines.

We next examined cell proliferation after treating the cells for several days with LiCl or alsterpaullone at approximately the LD_50 _dose. For MEFs and HCT116 cells, we saw a constant increase in cell number with time. This increase was, though, much weaker in the presence of LiCl or alsterpaullone (Figure 1B I-IV). For U2OS cells, we observed a slightly different picture. Here we found that after an initial increase in cell number, even in the presence of LiCl or alsterpaullone, the number of cells remained more or less constant (alsterpaullone), or even declined (LiCl; Figure 1B V). For SaOs-2, we observed a strong reduction in proliferation at initial time points, but at later time points inhibition of proliferation ceased (Figure 1B VI).

### LiCl induces cell death in p53-positive and p53-negative cells

**Figure 2:**
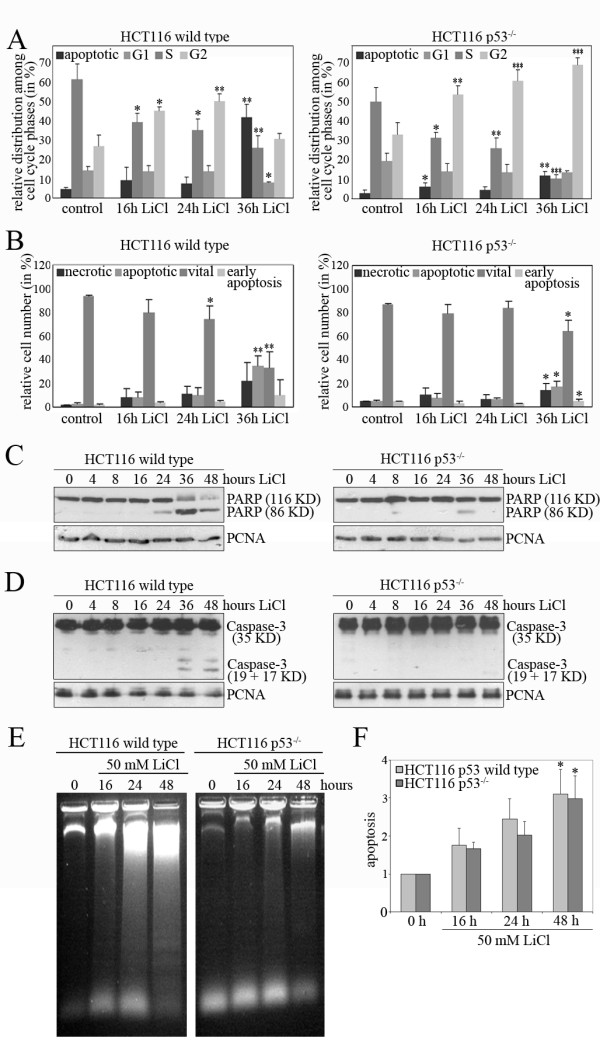
**LiCl induces cell death in p53-replete and p53-deficient cells**. (A) p53-positive and negative HCT116 cells were treated with 50 mM LiCl. Cells were fixed after 0, 16, 24 and 48 hours, stained with Draq5 and subjected to FACS analysis. Relative numbers of apoptotic cells and of cells in G1-, S- and G2-phase were determined. Mean values and standard deviations of three independent experiments were calculated and plotted. Statistical analysis was performed for alterations in the cell cycle and for induction of apoptosis in comparison to mock-treated cells (*: P < 0.05; **: P < 0.01; ***: P < 0.001). **(B) **p53-positive and negative HCT116 cells were treated with 50 mM LiCl. for 0, 16, 24 and 48 hours. Cells were stained with PI and FITC-coupled Annexin V, subjected to FACS analysis and relative numbers of apoptotic, necrotic and vital cells were determined. Mean values and standard deviations of three independent experiments were calculated and plotted. Statistical analysis was performed for alterations in the number of apoptotic, necrotic and vital cells in comparison to mock-treated cells (*: P < 0.05; **: P < 0.01). **(C) **HCT116 wild type and HCT116 p53^-/- ^cells were incubated with 50 mM LiCl for the indicated time. Cell lysates were prepared and 50 μg of protein were separated on a 10% SDS-PAGE gel. Proteins were transferred onto a PVDF membrane and probed with an antibody directed against PARP, and against PCNA for loading control. **(D) **HCT116 wild type and HCT116 p53^-/- ^cells were incubated with 50 mM LiCl for the indicated time. Cell lysates were prepared and 50 μg of protein were separated on a 15% SDS-PAGE gel. Proteins were transferred onto a PVDF membrane and probed with an antibody directed against Caspase-3, and against PCNA for loading control. **(E) **HCT116 wild type and HCT116 p53^-/- ^cells were incubated for the indicated times with 50 mM LiCl. Fragmented DNA was isolated and separated on a 1.4% agarose gel. **(F) **HCT116 wild type and HCT116 p53^-/- ^cells were incubated for the indicated times with 50 mM LiCl. The relative amount of apoptosis was determined using the Cell Death ELISA Plus kit (Roche). Mean values and standard deviation of three independent experiments were calculated and plotted. The readings for untreated cells were set to 1. Statistical analysis was performed for alterations in the number of apoptotic, necrotic and vital cells in comparison to mock-treated cells (*: P < 0.05).

We next investigated whether the decrease in proliferation after treatment of tumour cells with LiCl was due to the induction of cell death. By performing FACS-analysis, we observed both in p53-positive and in p53-negative HCT116 cell lines a clear increase in the sub-G1 peak starting at 16h after LiCl addition and increasing thereafter. This increase in the sub-G1 peak was more prominent in the p53-proficent cell line (Figure 2A). At the same time, we observed a significant decrease in G1 and S-phase cells and an increase in G2 cells, which was transient in the case of p53-replete cells and persistent in the case of p53-deficient cells (Figure 2A). Interestingly, although HCT116-p53-replete and p53-deficient cells both induced cell death in response to LiCl to a similar extent, they responded somewhat differently to the death-inducing stimulus. Both cell lines differed significantly regarding the alterations in G1-, S-phase and G2-cells. Annexin V/PI-staining revealed that there is also an increase in the number of necrotic cells in response to LiCl, although the values only reached statistical significance in the case of p53-negative cells at 36h after LiCl addition (Figure 2B).

Cell death by apoptosis is characterized by cleavage of PARP and Caspase-3, and by DNA fragmentation [24,25]. Consistent with the data from the FACS analysis, which indicated already that LiCl induces apoptosis, we observed a decrease in the 116 kDa form and an increase in the 86 kDa form of PARP after addition of LiCl in a time and dose-dependent manner (Figure 2C; Additional file 3, Figure S3A). In HCT116 wild type cells, the 86 kDa form of PARP was already detectable at twenty-four hours after LiCl treatment and most prominent at thirty-six hours post LiCl addition. Thereafter, both the 116 kDa and the 86 kDa form of PARP declined. Twelve hours after the initial signs of PARP cleavage, cleavage of Caspase-3 could be observed (Figure 2D; Additional file 3, Figure S3A). For cells deficient in p53, cleavage of PARP and Caspase-3 was much weaker and could only be observed at later time points, for example cleavage of PARP after 36 hours, and cleavage of Caspase-3 after 48 hours Figure 2C, D). This cleavage of PARP and Caspase-3 was clearly detectable when HCT116 cells had received a dose of 30 mM LiCl. P53-deficient cells showed PARP cleavage after a dose of 30 mM LiCl, while cleavage of Caspase-3 was already visible after a dose of 15 mM LiCl (Additional file 3, Figure S3A). However, despite this indication that p53 might be important for Caspase-3 cleavage after LiCl-treatment, we did not see reduced cleavage of Caspase-3 when we inhibited the transactivation function of p53 by pifithrin-α [23], the mitochondrial activities of p53 by pifithrin-μ [26], nor both activities by addition of both drugs (Additional file 3, Figure S3B). Downregulation of p53 by siRNA also had no strong impact on cleavage of Caspase-3 after treatment of U2OS cells with LiCl (Additional file 3, Figure S3C) Consistent with these observations, we found that chromosomal DNA was cleaved in p53-positive and p53-negative HCT116 cells. DNA fragmentation could already be observed at sixteen hours after LiCl addition and increased during the following eight hours (Figure 2E, F). In the absence of p53, DNA fragmentation was somewhat reduced, further supporting a modifying but facultative role of p53 for induction of cell death by LiCl.

### Inhibition of GSK-3 induces apoptosis in tumour cells

**Figure 3:**
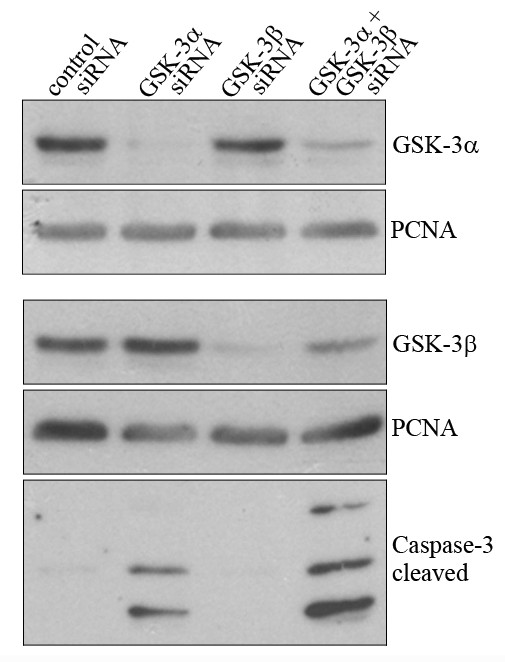
**Downregulation of GSK-3 leads to Caspase-3 activation**. U2OS cells were transfected with siRNA directed against GSK-3α, GSK-3β or both. 72 hours after transfection, cells were harvested and probed for GSK-3α and GSK-3β expression and for cleaved Caspase-3 by Western Blotting. Hybridisation with an antibody targeted against PCNA served as loading control.

The similar outcome after treatment of the tumour cell lines with the two inhibitors of GSK-3, LiCl and alsterpaullone suggested that the growth suppressive activities of LiCl in tumour cells may be due to inhibition of GSK-3. To substantiate this notion we determined whether downregulation of GSK-3α, GSK-3β or both isoforms by siRNA is sufficient to induce apoptosis as measured by Caspase-3 cleavage. As shown in Figure 3, siRNAs targeted against the two GSK-3 isoforms led to a strong reduction in GSK-3α and GSK-3β protein levels. At the same time, Caspase-3 cleavage was strongly increased after downregulation of GSK-3α, and this cleavage of Caspase-3 was further enhanced after downregulation of both GSK-3 isoforms (Figure 3). Transfection with a non-related control siRNA did not cause cleavage of Caspase-3, demonstrating the specificity of the effect for GSK-3 siRNAs

### LiCl induces cell death by the extrinsic apoptosis pathway

**Figure 4:**
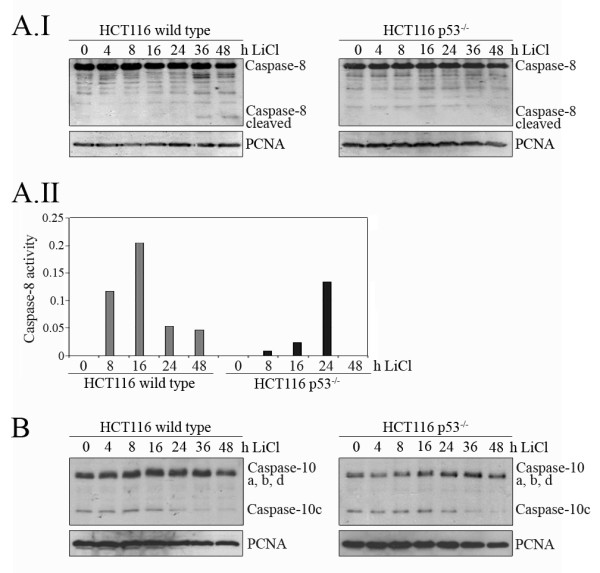
**LiCl activates the extrinsic apoptosis pathway**. HCT116 wild type and HCT116 p53^-/- ^cells were incubated with 50 mM LiCl for the indicated time. **(A.I) **Cell lysates were prepared and 50 μg of protein were separated on a 10% SDS-PAGE gel. Proteins were transferred onto a PVDF membrane and probed with an antibody directed against Caspase-8, and against PCNA for loading control. **(A.II) **Cell lysates were prepared and the activity of Caspase-8 was determined with the Caspase-8 activity assay (Clonetech). **(B) **Cells were lysed and 50 μg of protein were separated on a 10% SDS-PAGE gel. Proteins were transferred onto a PVDF membrane and probed with an antibody directed against Caspase-10, and against PCNA for loading control.

Apoptosis can be initiated by different signalling cascades (reviewed in: [27]). The most frequently used ones are the intrinsic pathway that is characterized by release of cytochrom C from mitochondria and activation of Caspase-9 and the extrinsic pathway that activates Caspase-8 and/or Caspase-10. To investigate which pathway is activated by LiCl-dependent cell death, we determined the release of cytochrome C. However, we failed to observe a significant increase in the amount of cytochrome C in the cytoplasm of LiCl-treated cells (Additional file 4, Figure S4). Likewise, we observed minor activation of Caspase-9, and only in some cell lines (data not shown). In contrast, Caspase-8 was strongly activated in p53 wild type cells, and to a lesser degree in HCT-116 cells with a genetic deletion of the p53 gene (Figure 4A). Similarly, Caspase-10 and in particular Caspase-10c became cleaved after treatment of cells with LiCl in a time and dose dependent manner (Figure 4B; Additional file 3, Figure S3A).

**Figure 5:**
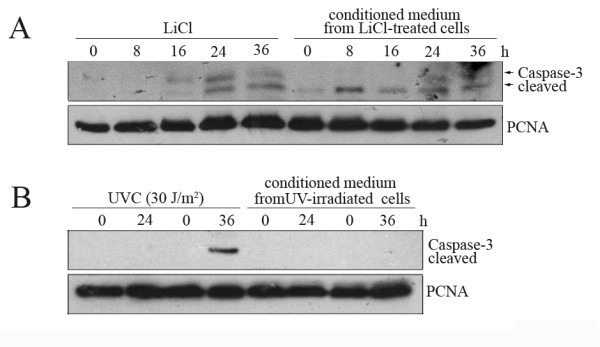
**LiCl stimulates the release of a cell death-mediating factor**. (A) U2OS cells were treated with 50 mM LiCl. After 24 hours, the medium was replaced and cells were incubated for further 8, 16, 24 or 36 hours. Cells were harvested and cell lysates were prepared (LiCl). The conditioned medium from these cells was collected after 8, 16, 24 or 36 hours and transferred to untreated U2OS cells. Thereafter, these cells were incubated for an additional 24 hours. Cell lysates were prepared and 50 μg of protein were separated on a 15% SDS-PAGE gel. Proteins were transferred onto a PVDF membrane and probed with an antibody directed against cleaved Caspase-3, and against PCNA for loading control. **(B) **U2OS cells were irradiated with 30 J/m^2 ^UVC. After 24 hours, conditioned medium was collected and transferred onto fresh U2OS cells. The cells were harvested ("0") or incubated for a further 24 hours or 36 hours. Cells that received the conditioned medium were harvested immediately ("0") or incubated for further 24 or 36 hours. Cell lysates were prepared and 50 μg of protein were separated on a 15% SDS-PAGE gel. Proteins were transferred onto a PVDF membrane and probed with an antibody directed against cleaved Caspase-3, and against PCNA for loading control.

Activation of Caspase-8 and -10 and absence of cytochrome C release strongly suggested that treatment of cells with LiCl initiated the extrinsic apoptosis pathway. This pathway is commonly activated by binding of soluble ligands to death receptors (reviewed in [28]). We therefore speculated that treatment of cells with LiCl leads to the release of a soluble factor that binds to death receptors. To test this notion, we transferred conditioned medium from LiCl-treated cells to untreated cells and investigated initiation of cell death by determining Caspase-3 cleavage. Indeed, similar to cells that had received LiCl, cells that had received conditioned medium from LiCl-treated cells also showed cleavage of Caspase-3 (Figure 5A). This initiation of cell death by conditioned medium was specific to LiCl-treated cells as, for example, UVC-treated cells, which also showed cleavage of Caspase-3, did not secrete a Caspase-3-activating factor into the culture medium (Figure 5B).

**Figure 6:**
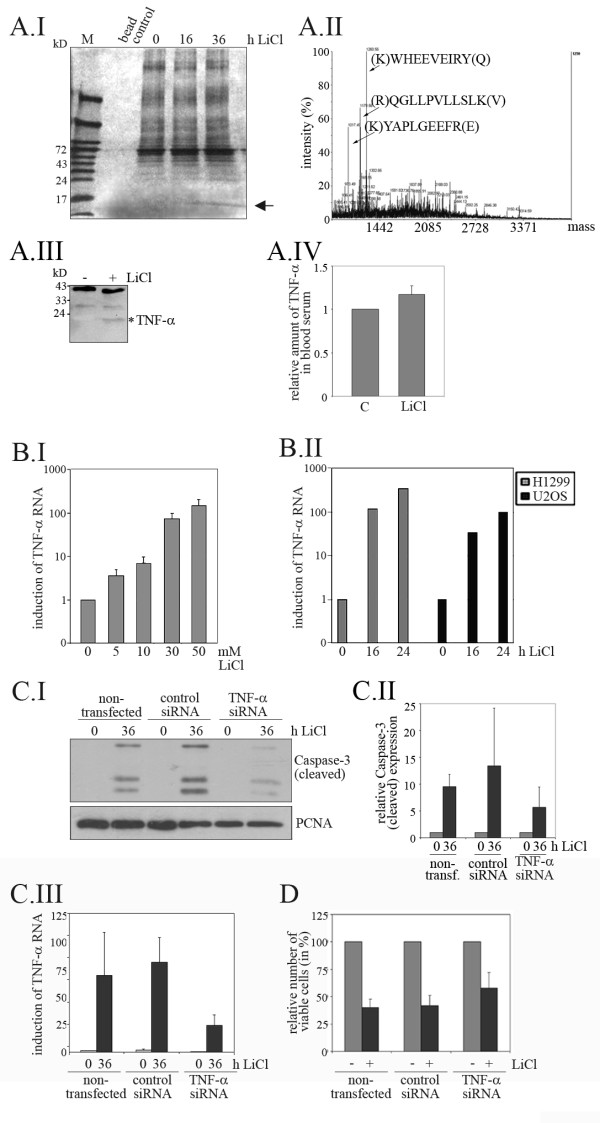
**LiCl stimulates the release of a TNF-α**. **(A.I) **U2OS cells were cultured in 0.5% FCS and stimulated with 50 mM LiCl. At the indicated times, the culture medium was harvested and proteins were precipitated using Strataclean Resin. Proteins were eluted with sample buffer and separated on a 15% SDS-PAGE gel. The gel was silver-stained and photographed. The arrow points to a protein that is only present in LiCl treated cells. **(A.II) **The protein that was only present in the cell culture supernatant after treatment of cells with LiCl was eluted from the gel, digested with trypsin and subjected to MALDI-TOF sequencing. The graph shows the spectrogram from MALDI-TOF sequencing. Arrows point to peptides characteristic of TNF-α. **(A.III) **U2OS cells were cultured in 0.5% FCS and stimulated with 50 mM LiCl. After 36 hours, the culture medium was harvested and proteins were precipitated using Strataclean Resin, eluted with sample buffer and separated on a 15% SDS-PAGE gel. Proteins were transferred onto a PVDF membrane and probed with antibodies directed against TNF-α. **(A.IV) **Three Wistar rats were injected with 50 mg/kg of LiCl or with the vehicle (PBS) for 4 weeks. From 3 weeks after the first drug dose, blood was collected twice a week and TNF-α levels were determined in the serum using a rat-TNF-α ELISA kit (Invitrogen). Mean values and standard deviations were calculated from three bleedings and blotted. The value for rats that received the vehicle was set to 1. **(B.I) **U2OS cells were treated with the indicated concentrations of LiCl for 24 hours. RNA was prepared and the amount of TNF-α RNA was determined by qRT-PCR. Mean values and standard deviation of 2^ΔCT ^values of 3 independent experiments were plotted. The data for untreated cells were set to 1. **(B.II) **U2OS and H1299 cells were treated with 50 mM LiCl and harvested at the indicated time points. RNA was prepared and the amount of TNF-α RNA was determined by qRT-PCR. Mean values of 2^ΔCT ^values of 2 (H1299) and 3 (U2OS) independent experiments were plotted. The data for untreated cells were set to 1. **(C) **U2OS were transfected with siRNA targeted against TNF α or with a control siRNA, or left untransfected for control. 24 hours after transfection, 50 mM LiCl were added and the cells were incubated for a further 36 hours. Cells were harvested and divided into two aliquots. One of the aliquots was lysed and 50 μg of protein were separated on a 15% SDS-PAGE gel. Proteins were transferred onto a PVDF membrane and probed with antibodies directed against cleaved Caspase-3, and against PCNA for a control (C.I). Mean values and standard deviations from three independent experiments were calculated and plotted. Values for untreated cells were set to 1 (C.II). From the second aliquot, RNA was prepared and the amount of TNF-α RNA was determined by qRT-PCR. Mean values and standard deviations of 2^ΔCT ^values of the three experiments were calculated and plotted. The values obtained for TNF-α RNA in the absence of LiCl were set to 1 (C.III). **(D) **U2OS cells were transfected with siRNA targeted against TNF-α or with a control siRNA, or left untransfected for control. 24 hours after transfection, 50 mM LiCl were added and the cells were incubated for a further 36 hours. Relative numbers of viable cells were determined by MTT-assay.

In order to identify the secreted factor, we precipitated the proteins in the cell culture supernatant of LiCl-treated and of non-treated cells, separated these proteins on a SDS-PAGE gel and stained the gel. We specifically and repeatedly observed a protein band of the size of about 17 kDa in LiCl treated cells at 36 hours after LiCl addition that was absent in the non-treated control or in cells treated with the same dose of LiCl, but harvested at sixteen hours after LiCl addition (Figure 6A.I). Sequencing of this protein revealed it to be TNF-α (Figure 6A.II). Furthermore, a protein of about 17 kD was specifically recognized by an anti-TNF-α antibody in the supernatant of LiCl-treated U2OS cells, but not in control cells (Figure 6A.III). Moreover, rats that had received a daily dose of LiCl for three to four weeks had higher levels of TNF-α in their serum than vehicle-treated controls (Figure 6A.IV).

To further support the notion that TNF-α expression is induced by LiCl, we determined TNF-α RNA levels in LiCl-treated and untreated cells. As shown in Figure 6B we observed a strong dose- and time-dependent increase in TNF-α RNA after LiCl treatment. This increase in TNF-α RNA was observed for both p53-positive (U2OS) and p53-negative (H1299) cells (Figure 6B.II).

TNF-α is a potent inducer of cell death in both U2OS and HCT116 cells (Additional file 5, Figure S5A). To determine whether TNF-α is required for LiCl-induced cell death, we knocked down TNF-α in U2OS and HCT116 cells prior to the addition of LiCl, then harvested the cells thirty-six hours after LiCl addition and determined Caspase-3 cleavage. Most importantly, downregulation of TNF-α by siRNA reduced Caspase-3 cleavage after LiCl-treatment by about 50% (Figure 6C.I and II; Additional file 5, Figure S5B) and reduced TNF-α RNA induction after treatment of the cells with LiCl by more than 60% (Figure 6C.III). Furthermore, transfection of U2OS cells with TNF-α siRNA strongly increased the number of cells that survived a 50 mM LiCl treatment (Figure 6D). In summary, these data strongly support the notion that TNF-α is an important mediator of apoptosis induction by LiCl.

**Figure 7:**
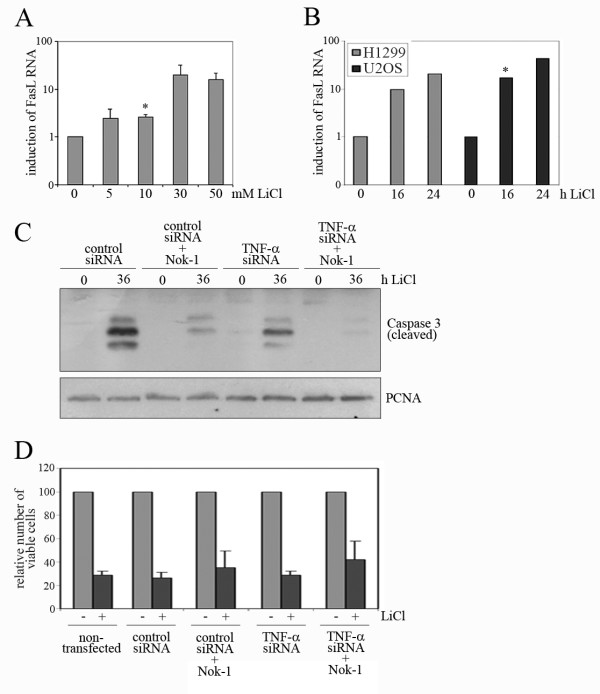
**LiCl induces FasL**. **(A) **U2OS cells were treated with the indicated concentrations of LiCl for 24 hours. RNA was prepared and the amount of FasL RNA was determined by qRT-PCR. Mean values and standard deviation of 2^ΔCT ^values of 3 independent experiments were plotted. The data for untreated cells were set to 1. **(B) **U2OS and H1299 cells were treated with 50 mM LiCl and harvested at the indicated time points. RNA was prepared and the amount of FasL RNA was determined by qRT-PCR. Mean values of 2^ΔCT ^values of 2 (H1299) and 3 (U2OS) independent experiments were plotted. The data for untreated cells were set to 1. **(C) **HCT were transfected with siRNA targeted against TNF-α or with a control siRNA. 20 hours after transfection, Nok-1 antibody was added at a dilution of 1:500 where indicated. Four hours later, 50 mM LiCl were added and the cells were incubated for a further 36 hours. Cells were lysed and 50 μg of protein were separated on a 15% SDS-PAGE gel. Proteins were transferred onto a PVDF membrane and probed with antibodies directed against Caspase-3, or against PCNA for a control. **(D) **U2OS cells were transfected with siRNA targeted against TNF-α or with a control siRNA, or left untransfected for control. 20 hours after transfection, Nok-1 antibody was added where indicated at a dilution of 1:500. Four hours later, 25 mM LiCl were added and the cells were incubated for a further 36 hours. Relative numbers of viable cells were determined using the MTT-assay.

Beside TNF-α, we also determined alterations in FasL expression, another protein that is able to form a DISC complex and to induce apoptosis by the extrinsic pathway (reviewed in: [5]. We found that FasL RNA was also strongly induced by LiCl (Figure 7A, B), albeit not so robustly as the induction of TNF-α RNA (Figure 6B). When we inhibited binding of FasL to its receptor by pre-incubation of LiCl-treated cells with the anti-FasL antibody Nok-1, cleavage of Caspase-3 was strongly reduced (Figure 7C; Additional file 5, Figure S5C) and cell survival was reproducibly upregulated (Figure 7D). Moreover, when we combined downregulation of TNF-α and blocking of FasL/Fas interaction, we observed an even greater reduction in Caspase-3 cleavage (Figure 7C; Additional file 5, Figure S5C) and an additive effect on cell survival (Figure 7D), clearly implicating both TNF-α and FasL as mediators of LiCl-induced cell death.

### Regulation of apoptosis by pro-and anti-apoptotic factors after LiCl treatment

**Figure 8:**
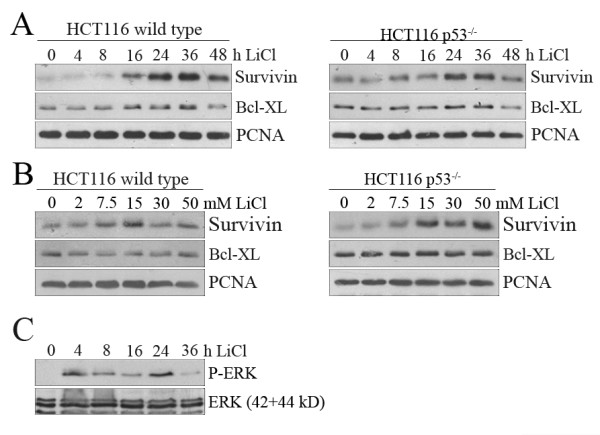
**LiCl increases the amount of Survivin and of phosphorylated ERK**. **(A) **HCT116 wild type and HCT116 p53^-/- ^cells were incubated with 50 mM LiCl for the indicated times. Cell lysates were prepared and 50 μg of protein were separated on a 10% SDS-PAGE gel. Proteins were transferred onto a PVDF membrane and probed with antibodies directed against Survivin and Bcl-XL, and against PCNA for a loading control. **(B) **HCT116 wild type and HCT116 p53^-/- ^cells were incubated with the indicated concentrations of LiCl for 48 hours. Cell lysates were prepared and 50 μg of protein were separated on a 10% SDS-PAGE gel. Proteins were transferred onto a PVDF membrane and probed with antibodies directed against Survivin and Bcl-XL, and against PCNA for a loading control. **(C) **U2OS cells were treated with 50 mM LiCl and harvested at the indicated time points. Cell extracts were prepared and 50 μg of protein were separated on a 10% SDS-PAGE gel. Proteins were transferred onto a PVDF membrane and probed with an antibody directed against phosphorylated ERK. The membrane was stripped and reprobed with an antibody directed against the p42 and p44 kDa forms of ERK, for a loading control.

To investigate the regulation of pro- and anti-apoptotic proteins that might be involved in the induction of apoptosis, we determined the amount of Survivin, Bcl-XL, Bid, XIAP and Bax proteins, and determined phosphorylation of extracellular signal-regulated kinase (ERK) after LiCl treatment of tumour cells. To determine whether any of these pro- and anti-apoptotic proteins are regulated by treatment of cells with LiCl, we added LiCl to the cell culture and harvested the cells at various time points. Surprisingly, the anti-apoptotic protein Survivin was induced by LiCl, although LiCl is clearly a potent inducer of cell death. Starting from sixteen hours after LiCl addition, we observed a significant increase in the amount of Survivin that was further increased up to thirty-six hours both in HCT116 wild type and p53-deficient cells (Figure 8A). This increase in the amount of Survivin was already evident from a dose of 15 mM LiCl on, yet decreased at higher doses in p53 wild type cells. In p53-deficient cells, we also observed an increase in Survivin from 15 mM on (Figure 8B). However in contrast to wild type cells, no decline was visible up to 50 mM LiCl, The amount of Bcl-XL, Bid, Bax and XIAP proteins remained unchanged (Figure 8A, B; Additional file 6, Figure S6). Beginning at four hours after LiCl treatment, we also observed a strong phosphorylation of p42-ERK that remained high for twenty-four hours and declined thereafter (Figure 8C).

### LiCl induces apoptosis in tumours of syngeneic rats

Induction of apoptosis by inhibition of GSK-3 offers the possibility of inducing cell death in tumour cells in a non-genotoxic way. We therefore investigated whether LiCl reduces tumour growth *in vivo*. To this end we employed the rat MT450 syngeneic mammary tumour model [29]. This cell line is routinely used in tumour growth and metastasis experiments in vivo in our institute, and its growth and other characteristics are well documented. Moreover, the use of a syngeneic animal model obviates problems associated with the growth of xenografts in immuno-compromised animals.

**Figure 9:**
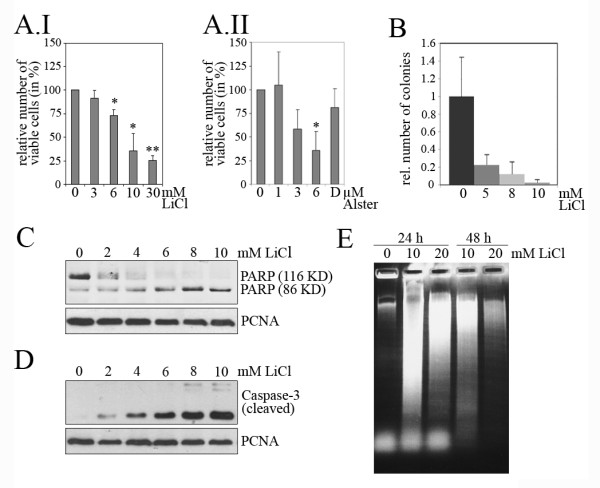
**LiCl induces apoptosis in MT450 breast cancer cells**. **(A) **MT450 rat mammary tumour cells were plated in 96-well plates at a density of 1 × 10^3 ^cells per well. 24 hours after plating, LiCl and alsterpaullone were added to the indicated concentrations. 72 hours after drug addition, the relative amount of viable cells was determined using the MTT-assay. Mean values and standard deviations of three independent experiments were calculated and plotted. The relative number of cells in the absence of drug was set to 100% (D: DMSO control). Statistical analysis was performed for alterations in relative cell proliferation in comparison to mock-treated cells (*: P < 0.05; **: P < 0.01). **(B) **1 × 10^3 ^MT450 cells were mixed with medium and methylcellulose 4000 containing the indicated doses of LiCl. After two weeks, the number of colonies was determined. The graph shows mean values and standard deviation of three independent experiments. The number of colonies in the absence of LiCl was set to 1. **(C) **MT450 cells were incubated for 36 hours with the indicated doses of LiCl. Cell lysates were prepared and 50 μg of protein were separated on a 10% SDS-PAGE gel. Proteins were transferred onto a PVDF membrane and probed with an antibody directed against PARP, and against PCNA for a loading control. **(D) **MT450 cells were incubated with the indicated doses of LiCl for 36 hours. Cell lysates were prepared and 50 μg of protein were separated on a 15% SDS-PAGE gel. Proteins were transferred onto a PVDF membrane and probed with an antibody directed against Caspase-3, or against PCNA for a loading control. **(E) **MT450 cells were incubated with 10 or 20 mM LiCl for 24 or 48 hours or left untreated for a control. Fragmented DNA was isolated and separated on a 1.4% agarose gel.

Prior to animal experiments, we tested the response of the MT450 rat mammary tumour cells to LiCl and alsterpaullone. As shown in Figure 9A, LiCl and alsterpaullone strongly reduced the number of viable MT450 cells in a dose dependent manner as assessed by the MTT assay (Figure 9A). Likewise, LiCl strongly reduced the colony forming ability of MT450 cells (Figure 9B). The reduction in proliferation and colony number was accompanied by cleavage of PARP and Caspase-3, and by DNA fragmentation in cell culture experiments (Figure 9C, D, E), indicating that MT450 cells respond to LiCl treatment by undergoing apoptosis in a similar manner to the other cell lines investigated in this study.

**Figure 10:**
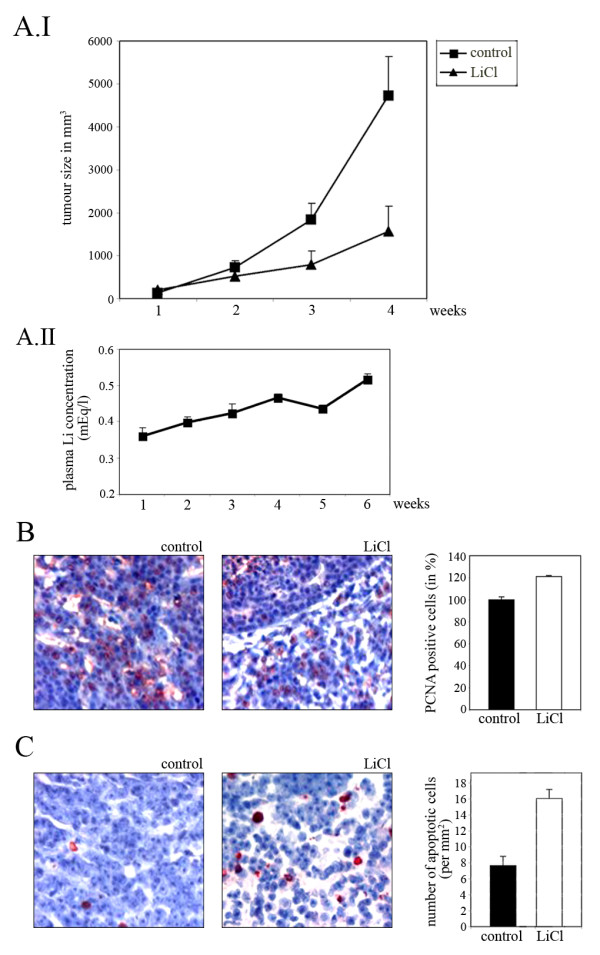
**LiCl reduces tumour growth in syngeneic rats**. **(A) **Wistar rats were injected with 50 mg/kg of LiCl dissolved in PBS or received the same amount of PBS without LiCl for control. 14 days after the first drug dose, 1 × 10^5 ^MT450 cells were inoculated subcutaneously. Tumour sizes and lithium concentrations in the blood were determined once a week. The graphs show mean values for 6-8 individuals (A.I) and corresponding blood serum concentrations of lithium (A.II). **(B) **Tumours were fixed and sections were stained for PCNA expression. PCNA-positive cells of an area of 25 mm^2 ^were counted. The graph shows the mean values and standard deviations of PCNA-positive cells (in%). **(C) **Tumour sections were stained for apoptotic cells. TUNEL-positive cells in an area of 25 mm^2 ^were counted. The graph shows the mean values and standard deviations of TUNEL-positive cells.

To determine whether inhibition of GSK-3β has an effect on tumour growth *in vivo*, we implanted MT450 cells into syngeneic rats and examined the effect of LiCl on the outgrowth of the ensuing tumours. One week prior to transplantation of tumour cells, we started to inject a LiCl solution into a group of eight Wistar Furth rats once a day. For controls, a similar group of animals was left untreated. Blood was regularly taken from the animals and monitored for levels of lithium. When the serum concentration of lithium reached a concentration of 0.4 mEq/l, we inoculated 5 × 10^6 ^MT450 cells into each group of rats. Continued daily injections thereafter with LiCl led to a sustained increase in serum concentrations of lithium to over 0.5 mEq/l (Figure 10A.II).

Tumour growth was regularly monitored. In all groups of animals, tumours developed with 100% penetrance. However, as can be seen in Figure 10A.I, animals receiving LiCl injections exhibited a substantial and significant reduction in tumour growth compared to control animals. The lithium treatment did not result in any obvious signs of toxicity in the experimental animals, such as weight loss or other symptoms associated with high serum lithium levels.

When tumours in control rats reached a size of about 500 mm^3^, we sacrificed the animals, and analyzed the tumours histologically. We stained tumour sections with an antibody directed against proliferating cell nuclear antigen (PCNA) and counted PCNA-positive cells to determine the proliferative capacity of the tumour. In addition, we performed TUNEL-assays to determine how many tumour cells underwent apoptosis.

Tumour sections from rats treated with LiCl or non-treated for control showed an relatively equal number of PCNA-positive cells (Figure 10B). However, when we stained tumour sections to label apoptotic cells, the number of TUNEL-positive cells was significantly increased in sections of tumours derived from LiCl-treated rats (Figure 10C).

## Discussion

In this study, we explored the effect of LiCl on proliferation and survival of tumour cells. We found that LiCl induced apoptosis not only in cell culture, but also in tumour cells *in vivo*, as demonstrated in syngeneic animal models treated with non-toxic concentrations of LiCl.

Our experiments demonstrate that LiCl and alsterpaullone, two widely used inhibitors of GSK-3 activity, are both able to induce apoptosis of tumour cells. These compounds inhibit GSK-3 by different mechanisms. Paullones were initially identified as CDK1/CDK2/CDK5 inhibitors using the COMPARE analysis of a data-base of compounds tested in the National Institute of Health. A structure/activity relationship study led to the development of the more potent CDK inhibitors kenpaullone and alsterpaullon. These proved to also be excellent GSK-3 inhibitors and in fact inhibit GSK-3 about ten-fold more potently than they do CDK1 [30]. Lithium is a non-competitive inhibitor of GSK-3 and its effect is reversible *in vitro*. It potently inhibits GSK-3 (K_i _= 2 mM), but is not a general inhibitor of other protein kinases [7]. Lithium has also been reported to inhibit inositol-monophosphatase, phospho-monoesterases and phospho-glucomutase [31-33], but these off-target effects require higher doses of the compound.

Treatment of cells with lithium inhibits GSK-3-dependent phosphorylation of the microtubule-associated protein Tau and induces accumulation of cytoplasmic Armadillo/β-Catenin in PC12 and Drosophila S2 cells, demonstrating that lithium can mimic Wingless signalling in intact cells [8], consistent with the notion that its major cellular effect is the inhibition of GSK-3. Moreover, downregulation of GSK-3 also resulted in cleavage of Caspase-3, supporting the idea that the apoptosis-inducing activity of LiCl is mediated by inhibiting GSK-3.

In the presence of the GSK-3 inhibitors, cell proliferation ceased and apoptosis was induced in all tumour cell lines that we investigated. Although in certain contexts GSK-3 has been found to act as a pro-apoptotic kinase (reviewed in: [34]), our results clearly show that tumour cells obviously require GSK-3 activity for cell survival. In the presence of GSK-3 inhibitors, cells were positive for all investigated features of apoptosis. Caspase-3 was processed into its active form, PARP was cleaved and DNA was fragmented into oligonucleosomes. At the same time, we did not observe any evidence for induction of senescence or cell death by autophagy (data not shown). Induction of cell death after inhibition of GSK-3 has also been observed by others [35-41].

By what molecular mechanism does LiCl induce apoptosis? GSK-3 has numerous cellular targets. For example, GSK-3 is well known as an important regulator of the canonical Wnt pathway (reviewed in: [42]). In unstimulated cells, GSK-3 phosphorylates the N-terminal domain of β-Catenin, thereby targeting it for ubiquitination and proteasomal degradation. Exposure of cells to Wnts leads to inactivation of GSK-3 and correspondingly to the accumulation of β-Catenin. However, this is unlikely to be involved in LiCl-mediated apoptosis as enhanced levels of β-Catenin have anti-apoptotic effects (e.g. [43]). Another important protein that is regulated by GSK-3 is Mdm2. GSK-3 phosphorylates the Mdm2 oncoprotein within its central domain at sites that are essential for Mdm2-mediated degradation of the p53 tumour suppressor protein. As a consequence, p53 accumulates in the absence of GSK-3 activity, which allows transcription of its target genes, among which are a large number of growth arrest- and apoptosis-inducing candidates [11,22]. However, despite the correlation of GSK-3 inhibition and p53 activation, we found that p53, if at all, played only a minor role in the induction of cell death after treatment of cells with LiCl as cells both with and without p53 underwent apoptosis in the presence of LiCl. Moreover, inhibition of p53 by drugs or downregulation did not reduce Caspase-3 cleavage or increase cell survival after treatment with LiCl. Nevertheless, cleavage of PARP and Caspase-3 as well as fragmentation of DNA was significantly reduced in p53-deficient HCT116 cells and cell death was initiated slightly earlier in the p53 positive cells. However, this may be a characteristic of the individual cell line. Although the p53-negative cell line HCT116 cell line is derived from the p53-positive one and should thus have the same genotype apart from p53, both cell lines may have acquired alterations later on, which may result in a different behaviour.

Our results show that a major apoptosis-inducing mechanism that is induced in both p53-positive and negative cells by LiCl is the production of TNF-α and FasL, two death-receptor ligands that activate the extrinsic pathway of apoptosis in an autocrine manner, as evidenced by activation of Caspase-8. These findings are consistent with two earlier reports in which TNF-α was found to be secreted from LiCl-stimulated monocytes [44,45]. Interestingly, earlier articles reported about a sensitizing and potentiating role of LiCl for TNF-α-mediated cytotoxicity [46,47]. We show that LiCl rather activates the same signalling cascade that TNF-α does rather than activating a second and complementary death signalling cascade.

TNF-α and FasL RNAs were strongly induced after LiCl treatment of both p53-negative H1299 and p53-positive U2OS cells. Importantly, downregulation of LiCl-induced TNF-α by siRNA or inhibition of FasL/Fas interaction by a blocking antibody reduced Caspase-3 cleavage and increased the relative number of viable cells. Nevertheless, protection from LiCl-induced apoptosis by siRNA-mediated inhibition of TNF-α expression or treatment with the Nok-1 antibody, even in combination, never reached 100%. A possible explanation for this observation might be that the robust induction of TNF-α and FasL by LiCl was so strong that it was impossible to suppress it completely by siRNA or antibody treatment. As shown in Figure 6C.III higher levels of TNF-α are still present in LiCl-treated cells compared to non-treated cells, even after transfection of siRNA, which further supports this notion. Alternatively or in addition, the observed phosphorylation of ERK, an activator of death-inducing kinase DAPK as well as other, not investigated factors, may have contributed to the initiation of apoptosis by LiCl after downregulation of TNF-α.

A major finding of this paper is that LiCl not only reduced cell proliferation in cell culture but also inhibited tumour growth in syngeneic animal models. We observed a significant increase in TUNEL-positive apoptotic cells in tumours from rats that were treated with LiCl, while we did not see a decrease in the number of PCNA positive proliferating cells. We therefore conclude that the reduction in tumour size in LiCl-treated animals is due to the induction of apoptosis. The steady-state level of lithium that we achieved in the animals is in the range of the concentration that is well tolerated by humans [48]. Additionally, we did not observe any loss in weight of the rats or other evidence of toxic effects. Moreover, we observed inhibition of cell proliferation and induction of apoptosis in cell culture experiments using significantly lower concentrations of lithium than those achieved in the animal experiments. Together these observations suggest that our experimental tumour data should be highly relevant and applicable to human cancer therapy.

Our data have several important implications regarding cancer therapy. Induction of cell death by GSK-3 was largely independent of p53, although the presence of p53 slightly enhanced the cell death-inducing activity of LiCl, particularly at low doses. Nevertheless, it should be noted that sequencing of the *p53 *gene in the MT450 breast tumour cell line revealed a mutation at position 174 within a hot spot of inactivating mutations, where tryptophan was replaced with a cysteine (data not shown). The positive response of the tumours in the rat to LiCl therefore further supports the p53-independence of apoptosis induction by LiCl. Considering that about 50% of tumours possess mutations in the *p53 *gene, inhibition of GSK-3 would therefore also be suitable for the treatment of p53-negative and -mutant tumours that are refractory to other types of treatment. However, it should be noted that two previous studies observed a strong p53-dependency for the inhibition of tumours in mice by inhibitors of GSK-3 [40,41]. The reason for this discrepancy is probably that while we treated the cells with LiCl, the other studies employed Purvalanol A and LY2119301 to inhibit GSK-3 [40,41]. It is possible that these compounds have off-target effects that induce p53-dependent apoptosis more potently than their effects on GSK-3 activity. This notion is supported by the fact that these authors report activation of Bax and release of cytochrome C [40] which we did not observe. On the other hand, it cannot be entirely excluded that LiCl may have some GSK-3-independent death-inducing activities. Inhibition of GSK-3 by lithium is a well-established therapy that has been used for many years for the treatment of mental disorders. Our data indicate that inhibition of GSK-3 activity deserves to be investigated further as a potential anti-cancer treatment.

## Conclusions

LiCl induced cell death in p53-replete and p53-deficient cells. Thus, induction of cell death by LiCl does not require the presence of wild type p53, although we cannot completely rule out the possibility that LiCl may activate two or more death pathways, one of which may involve p53 while the other(s) may not. Induction of cell death strongly depended on the inhibition of GSK-3 and on the production of TNF-α and FasL, which are released into the culture medium after treatment of cells with LiCl and which mediated cleavage of Caspases-3 -8 and -10.

## Methods

### Cell culture

U2OS cells, H1299 cells and HCT116 cells (wild type and p53^-/-^) were obtained from G. Taucher-Scholz (GSI, Darmstadt). Mouse embryonic fibroblasts (MEF; wild type and p53^-/-^) were provided by Bernd Kaina (University of Mainz) and RKO cells by Martin Scheffner (University of Konstanz). HaCat, Hela tk- and MT450 cells have been described previously [29,49,50]. All cell lines were cultured in DMEM supplemented with 10% FCS and 1% Penicillin/Streptomycin and incubated at 37°C and 5% CO_2 _in a humidified atmosphere.

Cell numbers were evaluated with an improved Neubauer chamber and a light microscope. To determine the number of dead cells, the cell suspension was mixed 1:1 with 1% trypan blue in PBS immediately prior to microscopy. LiCl was obtained from Sigma, alsterpaullone from Calbiochem, pifithrin-α and pifithrin-μ were purchased from Merck and TNF-α from Enzo Life Sciences.

For UV-irradiation, culture medium was removed and cells were washed once with PBS. After irradiation with 30 J/m^2 ^with a UVC lamp, the initial culture medium was added back and the cells were further incubated.

### Animal experiments

Young adult Wistar Furth rats weighing 200-250 g were kept on a 12 h light/dark cycle and fed ad libitum. Once a day, 50 mg/kg LiCl dissolved in PBS was injected i.p. Control animals received the same amount of PBS without LiCl. Two weeks after the first injection of LiCl, 5 × 10^6 ^MT460 cells in a total volume of 100 μl were inoculated subcutaneously. Tumour size was determined twice a week with a calliper. Blood samples were taken once a week six hours after LiCl treatment. Lithium concentration was determined by flame photometry by a commercial service provider (Laborgemeinschaft Albtal, Ettlingen, Germany)

For determining TNF-α serum levels, young adult Wistar Furth rats weighing 200-250 g were kept on a 12 h light/dark cycle and fed ad libitum. Once a day, 50 mg/kg LiCl dissolved in PBS was injected i.p. Control animals received the same amount of PBS without LiCl. Three weeks after the first injection of LiCl, blood samples were taken twice a week. TNF-α was determined in serum with a rat-TNF-α ELISA kit (Invitrogen) according to the manufacturer's recommendation.

The animal studies were approved by the local regulatory authority (Regierungspräsidium Karlsruhe). Permission #35-9185.81/G-83/04.

### Antibodies

Anti-PCNA antibodies were obtained from Dako (Immunohistochemistry) and from Santa Cruz (Western blotting). The anti-p53 antibodies DO-1 and Ab-2 were purchased from Santa Cruz and Merck, respectively. The anti-Bcl-XL, anti-Bax, anti-Caspase-3, anti-cleaved Caspase-8, anti-Caspase-8, anti-Survivin, anti-XIAP, anti-ERK and anti-GSK-3β antibodies were from Cell Signalling. The anti-GSK-3α anti-PARP, anti-Bid and anti-phosphorylated-ERK (p42 kDa) antibodies were from Santa Cruz. The anti-Caspase-10 antibody was purchased from MBL and the anti-cytochrome C and anti-FasL (Nok-1) antibodies were from BD Biosciences. HRP-coupled secondary antibodies were purchased from Dako.

### siRNA

siRNA targeted against GSK-3α (5'-CAUUCUCAUCCCUCCUCACUU-3'), GSK-3β (5'-GAGCAAAUCAGAGAAAUGAAC-3'), TNF-α (5'-GUGCUGGCAACCACUAAGA-3';) or p53 (AACCACUGGAUGGAGAAUA) or control siRNA (CCCCUUUUAAAAGGGGCCC) was transfected into U2OS or HCT116 cells using Lipofectamin (Invitrogen) to a final concentration of 50 pMol/ml according to the manufacturer's recommendations.

### Soft agar assay

1 × 10^3 ^MT450 cells were mixed with 1.5 ml DMEM medium supplemented with 10% FCS, 1% Penicillin/Streptomycin and 0.5 ml of a 1.2% solution of methylcellulose 4000 in DMEM. After two weeks, the number of colonies was determined with a light microscope.

### Colony assay

200 cells were plated per 5 cm-dish and cultured for two weeks in the presence or absence of LiCl. The culture medium was removed and cells were washed twice in ice cold PBS. Cells were fixed in methanol for 5 min, stained with 0.5% crystal violet in PBS and counted.

### MTT assay

2 × 10^4 ^cells per well were plated into a 96-well plate. 24 hours after plating, cells were stimulated with LiCl or alsterpaullone and cultured for an additional 3 days. Mock-treated controls were treated similarly. MTT was dissolved at a concentration of 1 mg/ml in DMEM supplemented with 10% FCS and 1% Penicillin/Streptomycin and added at a final concentration of 0.5 mg/ml for 4 hours. The medium was aspirated and 100 μl isopropanol were added. Formazan conversion was determined at 595 nm using an ELISA reader.

### Western blotting

Western blotting was performed as described previously [51]. To determine the release of cytochrome C, 5 × 10^6 ^cells were harvested, washed twice with ice-cold PBS and resuspended in 5 volumes of buffer A (150 mM sucrose, 20 mM HEPES pH 7.5, 1.5 mM MgCl_2_, 10 mM KCl, 1 mM EDTA, 1 mM EGTA, 1 mM DTT, 0.1 mM PMSF, 10 μg/ml leupeptin and 10 μg/ml aprotonin) and incubated for 20 min on ice. The lysate was cleared by centrifugation at 14 000 rpm for 15 minutes at 4°C. Mitochondria were pelleted by centrifugation at 100 000 rpm in a Sorvall Discovery ultracentrifuge for 1 hour at 4°C. 50 μg of the supernatant were mixed with an equal volume of 2 × sample buffer [51], heat denatured and loaded onto an SDS-PAGE gel.

### TUNEL assay and immunohistochemistry

Tumours were fixed overnight in formalin (3.7% formaldehyde in PBS), and stored in 50% ethanol until they were embedded in paraffin. Tumour sections were stained for apoptotic cells using the Apoptag kit (Chemicon) according to the manufacturer's recommendation. For PCNA staining, sections were deparaffinised, then incubated for 10 minutes in 2 M HCl and washed 4 times with H_2_O. Subsequently, sections were immersed in methanol/0.3% H_2_O_2 _for 20 minutes, washed 3 times with PBS, and then blocked for 15 minutes in PBS/10% rabbit serum. PCNA antibodies were diluted in PBS/10% rabbit serum to a final concentration of 10 μg/ml and incubated with the sections overnight at 4°C. The sections were washed 3 times with PBS and immersed in 3% H_2_O_2 _in PBS for 5 minutes. A biotinylated rabbit anti-mouse antibody, diluted 1:400 in PBS/10% FCS was applied to the sections and incubated for 30 minutes at room temperature. Sections were washed 3 times with PBS and incubated for 30 minutes with a StreptABComplex/HRP (Dako) solution, prepared according to the manufacturer's recommendations. Sections were washed 3 times with PBS, incubated with an AEC-one-component-solution (Innovative Diagnostic-Systems) for 10 min and counterstained with haematoxylin.

### Caspase-8 activity assay

Caspase-8 activity assay was performed according to the manufacturer's instructions (Clontech).

### FACS-analysis

For cell cycle analysis, cells were treated with 50 mM LiCl for 16, 24 and 36 hours, harvested, mixed with ice-cold 70% ethanol and fixed overnight at 4°C. Cells were pelleted at 530 g for 5 minutes, washed once with PBS and stained with Draq5 (Biostatus Ltd) at a final concentration of 10 μM for 15 minutes in the dark. DNA content of the cells was determined using a flow cytometer (FACScan; Becton Dickinson).

For assessing apoptosis/necrosis, cells were treated with 50 mM LiCl for 16, 24 and 36 hours, trypsinized, washed with PBS and resuspended in 400 μl Ca^2+^-binding buffer (10 mM HEPES pH 7.6, 140 mM NaCl, 5 mM CaCl_2_). Subsequently 1 μl of a 1 mg/ml propidium iodide solution (staining late apoptotic and necrotic cells) and 5 μl of FITC-coupled AnnexinV (BD Bioscience; to detect early apoptosis) were added to the cells. After incubation on ice for 10 min, cells were analyzed flow cytometrically (FACScan; Becton Dickinson).

### Determination of apoptosis by DNA fragmentation and Cell Death ELISA

For determining apoptosis by assessing DNA fragmentation, cells were lysed in 125 μl buffer A (20 mM EDTA, 50 mM Tris pH7.5, 1%NP40; pH7) for 1.5 min on ice and samples were centrifuged at 4500 rpm for 5 min. 10 μl of a 10% SDS-solution and 20 μl RNAse A (20 mg/ml) were added to the supernatant and incubated for 2 h at 56°C. Then 25 μl Proteinase K (10 mg/ml) were added and samples were incubated for 2 h at 37°C. 50 μl of ammonium acetate (10 M) and 500 μl EtOH were added, and the DNA was precipitated over night at -20°C before the DNA was analysed by agarose gel electrophoresis.

Induction of apoptosis and necrosis was furthermore determined using the Cell Death ELISA-Plus kit (Roche). The assay was performed according to the manufacturer's recommendations.

### Extraction of proteins with StrataClean Resin

5 μl of resin were washed with PBS and incubated with 5 ml of cell culture supernatant for 1 hour at room temperature. The resin was washed twice with PBS and bound proteins were eluted by boiling in 1 × sample buffer for 5 min at 95°C.

### Silver staining and MALDI-TOF Sequencing

The gel was fixed in 40% EtOH and 6% acetic acid for 30 min. Afterwards the gel was incubated in solution A (30% EtOH; 5% sodium thiosulfate; 6.8% sodium acetate) and washed 3 times for 5 min with H_2_O. The gel was then incubated in solution B (0.25% silver nitrate; 0.015% formaldehyde) for 20 min and washed twice for 1 min with H_2_O. The staining was developed in solution C (2.5% sodium carbonate; 0.0075% formaldehyde) and the reaction was stopped by gently shaking the gel in an EDTA solution (1.46% EDTA in H_2_O) for 10 min.

The protein of interest was cut out from the gel and destained twice for 10 min each in washing solution (10 mM K_4_Fe(CN)_6_, 66 mM S_2_O_3_). The gel piece was then washed for 10 min in 10 mM NH_4_HCO_3 _and incubated for 10 min in ACN solution (50% ACN, 5 mM NH_4_HCO_3_. To reduce disulfide bonds in the proteins, the gel piece was incubated for 15 min at 60°C in 10 mM DTT. Unreacted DTT was removed and free cysteine residues were alkylated by incubation of the gel pieces with IAA solution (0.1% iodacetamid in 10 mM NH_4_HCO_3_). The gel piece was washed 6 times for 10 min in NH_4_HCO_3 _and dried. Trypsin solution (100 ng/μl trypsin in 10 mM NH_4_HCO_3_) was added to the dried gel piece, overlaid with 10 mM NH_4_HCO_3 _and incubated overnight at RT with constant shaking. The digest was stopped by the addition of 0.1% TFA. Protein fragments were eluted by centrifugation and analysed by MALDI-TOF.

### RT-PCR

Total RNA was prepared from cells using the RNeasy kit (Qiagen) according to the manufacturer's instructions and treated with DNase I to remove residual genomic DNA. RNA was transcribed into cDNA using random primers and RevertAid™ H MinusM-MuLV reverse transcriptase (Fermentas). The quality of cDNA was tested by conventional PCR and actin primers. Real-time PCR was performed in duplicates with a SYBR Green PCR mixture (Qiagen). The cDNA was denatured for 4 min at 90°C followed by 40 cycles of 90°C for 30 s, 60°C for 45 sec and 72°C for 45 sec and a final extension step of 5 min at 72°C using the Step-One-Plus sequence detection system (Applied Biosystems) and gene specific primers. The signals were normalized to the signals for the ribosomal subunit 36B4. Sequences of primers are available on request.

### Statistical analysis

Differences between treated and mock-treated controls were examined using the unpaired students T-Test. P values < 0.05 were considered to be statistically significant. All of the statistical tests were performed with Microsoft Office Excel 2010 software.

## Competing interests

The authors declare that they have no competing interests.

## Authors' contributions

LK, GM and CB have carried out experiments. IN has performed the statistical analysis JS, WT and CO have helped with/supervised parts of the study. CB has initiated and supervised the whole study. CB and JS have written the manuscript.

All authors have read and approved the manuscript.

## Supplementary Material

Additional file 1 **Figure S1 - p53 status of cell lines**. HCT116 wild type and HCT116 p53^-/-^, mouse embryonic fibroblasts (MEF) wild type and MEF p53^-/-^, SaOs-2 and U2OS cells were lysed. 50 μg of protein were separated on a 10% SDS-PAGE gel and transferred onto a PVDF membrane. The membrane was probed with an antibody directed against p53, and against PCNA for loading control. Click here for file

Additional file 2 **figure S2 - The reduction in proliferation and colony forming ability in response to LiCl is independent of p53**. **(A) **U2OS and HCT116 wild type cells were plated in 96-well plates at a density of 1 × 10^3 ^cells per well. 20 hours after plating, pifithrin-α or the vehicle (DMSO) were added to the cells at a final concentration of 50 μM. After additional 4 hours, LiCl was added at the indicated concentrations. 72 hours after drug addition, the relative number of viable cells was determined by MTT-assay. Mean values and standard deviations of three independent experiments were calculated and plotted. The relative number of cells in the absence of drug was set to 100%. **(B) **U2OS cells were transfected with siRNA targeted against p53 or with a control siRNA. 24 hours after transfection LiCl was added at the indicated concentrations. 72 hours after drug addition, the relative number of viable cells was determined by MTT-assay. Mean values and standard deviations of three independent experiments were calculated and plotted. The relative number of cells in the absence of drug was set to 100%. **(C) **200 p53-positive (wild type) and p53-negative HCT116 (HCT116 p53^-/-^) cells were plated in 5 cm dishes. LiCl was added at the indicated doses. Two weeks after plating, colonies were stained with crystal violet and counted. The graph shows mean values and standard deviations of 3 independent experiments. Relative numbers of colonies of mock-treated cells were set to 100%. Click here for file

Additional file 3 **Figure S3 - PARP and Caspases are cleaved after treatment with LiCl**. (A) HCT116 wild type and HCT116 p53^-/- ^cells were incubated with the indicated doses of LiCl for 48 hours. Cell lysates were prepared and 50 μg of protein were separated on a 10% SDS-PAGE gel. Proteins were transferred onto a PVDF membrane and probed with an antibody directed against PARP, Caspase-3 and Caspase-10, or against PCNA for a loading control. **(B) **HCT116 wild type cells were incubated with 50 μM pifithrin-α, 10 μM pifithrin-μ, with both, or with vehicle (DMSO) for control. After 4 hours, 50 mM LiCl were added. 36 hours after the addition of LiCl, cell lysates were prepared and 50 μg of protein were separated on a 15% SDS-PAGE gel. Proteins were transferred onto a PVDF membrane and probed with an antibody directed against Caspase-3, or against PCNA for loading control. **(C) **U2OS cells were transfected with siRNA targeted against p53 or with a control siRNA. 24 hours after transfection 50 mM LiCl were added. 36 hours after the addition of LiCl, cell lysates were prepared and 50 μg of protein were separated on a 15% SDS-PAGE gel. Proteins were transferred onto a PVDF membrane and probed with an antibody directed against Caspase-3, or against PCNA for a loading control. Click here for file

Additional file 4 **Figure S4 - LiCl does not induce the release of Cytochrome C from mitochondria**. HCT116 wild type and HCT116 p53^-/- ^cells were incubated with 50 mM LiCl for the indicated time. Cells were lysed and mitochondria were separated from the cytoplasmic fraction by centrifugation. 50 μg of protein of the cytoplasmic fraction were separated on a 15% SDS-PAGE gel, transferred onto a PVDF membrane and probed with an antibody directed against cytochrome C, or PCNA for a loading control. Click here for file

Additional file 5 **Figure S5 - Caspase 3 cleavage and cell survival in dependence of TNF-α and FasL**. **(A) **U2OS and HCT116 wild type cells were plated in 96-well plates at a density of 1 × 10^3 ^cells per well. 24 hours after plating, TNF-α was added to the indicated concentrations. 72 hours after drug addition, the relative number of viable cells was determined by MTT-assay. Mean values and standard deviations of three independent experiments were calculated and plotted. The relative number of cells in the absence of TNF-α was set to 100%. **(B) **HCT116 wild type cells were transfected with siRNA targeted against TNF-α or with a control siRNA, or left untransfected for control. 24 hours after transfection, 25 mM LiCl were added and the cells were incubated for further 36 hours. Cells were lysed and 50 μg of protein were separated on a 15% SDS-PAGE gel. Proteins were transferred onto a PVDF membrane and probed with antibodies directed against cleaved Caspase-3, or PCNA for a control. **(C) **U2OS were transfected with siRNA targeted against TNF-α or with a control siRNA. 20 hours after transfection, Nok-1 antibody was added at a dilution of 1:500 where indicated. Four hours later, 50 mM LiCl were added and the cells were incubated for a further 36 hours. Cells were lysed and 50 μg of protein were separated on a 15% SDS-PAGE gel. Proteins were transferred onto a PVDF membrane and probed with antibodies directed against Caspase-3, or PCNA for a control. Click here for file

Additional file 6 **Figure S6 - S6: Treatment with LiCl does not affect expression of Bax, XIAP or Bid**. **(A) **HCT116 wild type and HCT116 p53^-/- ^cells were incubated with the indicated doses of LiCl for 48 hours. Cell lysates were prepared and 50 μg of protein were separated on a 10% SDS-PAGE gel. Proteins were transferred onto a PVDF membrane and probed with antibodies directed against Bax, XIAP and Bid, or against PCNA for a loading control. **(B) **HCT116 wild type and HCT116 p53^-/- ^cells were incubated with 50 mM LiCl for the indicated times. Cell lysates were prepared and 50 μg of protein were separated on a 10% SDS-PAGE gel. Proteins were transferred onto a PVDF membrane and probed with antibodies directed against Bax, XIAP and Bid, or against PCNA for a loading control. Click here for file
