# Supplementary figures and images for: LiCl induces TNF-α and FasL production, thereby stimulating apoptosis in cancer cells
Source: Cell Commun Signal. 2011 May 24;9:15. doi: 10.1186/1478-811X-9-15 (PMC3115922; doi:10.1186/1478-811X-9-15)

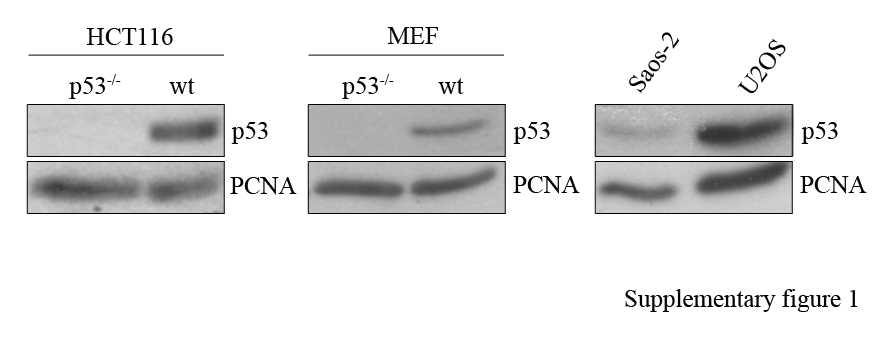

Supplement: Additional file 1 — Figure S1 - p53 status of cell lines. HCT116 wild type and HCT116 p53-/-, mouse embryonic fibroblasts (MEF) wild type and MEF p53-/-, SaOs-2 and U2OS cells were lysed. 50 μg of protein were separated on a 10% SDS-PAGE gel and transferred onto a PVDF membrane. The membrane was probed with an antibody directed against p53, and against PCNA for loading control. [file 1478-811X-9-15-S1.TIFF]

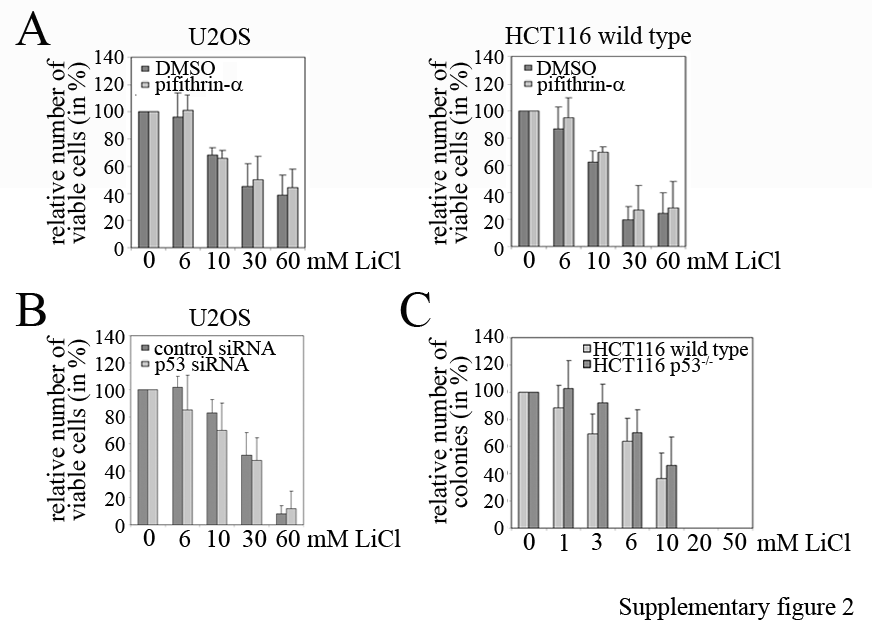

Supplement: Additional file 2 — figure S2 - The reduction in proliferation and colony forming ability in response to LiCl is independent of p53. (A) U2OS and HCT116 wild type cells were plated in 96-well plates at a density of 1 × 10^3^ cells per well. 20 hours after plating, pifithrin-α or the vehicle (DMSO) were added to the cells at a final concentration of 50 μM. After additional 4 hours, LiCl was added at the indicated concentrations. 72 hours after drug addition, the relative number of viable cells was determined by MTT-assay. Mean values and standard deviations of three independent experiments were calculated and plotted. The relative number of cells in the absence of drug was set to 100%. (B) U2OS cells were transfected with siRNA targeted against p53 or with a control siRNA. 24 hours after transfection LiCl was added at the indicated concentrations. 72 hours after drug addition, the relative number of viable cells was determined by MTT-assay. Mean values and standard deviations of three independent experiments were calculated and plotted. The relative number of cells in the absence of drug was set to 100%. (C) 200 p53-positive (wild type) and p53-negative HCT116 (HCT116 p53-/-) cells were plated in 5 cm dishes. LiCl was added at the indicated doses. Two weeks after plating, colonies were stained with crystal violet and counted. The graph shows mean values and standard deviations of 3 independent experiments. Relative numbers of colonies of mock-treated cells were set to 100%. [file 1478-811X-9-15-S2.TIFF]

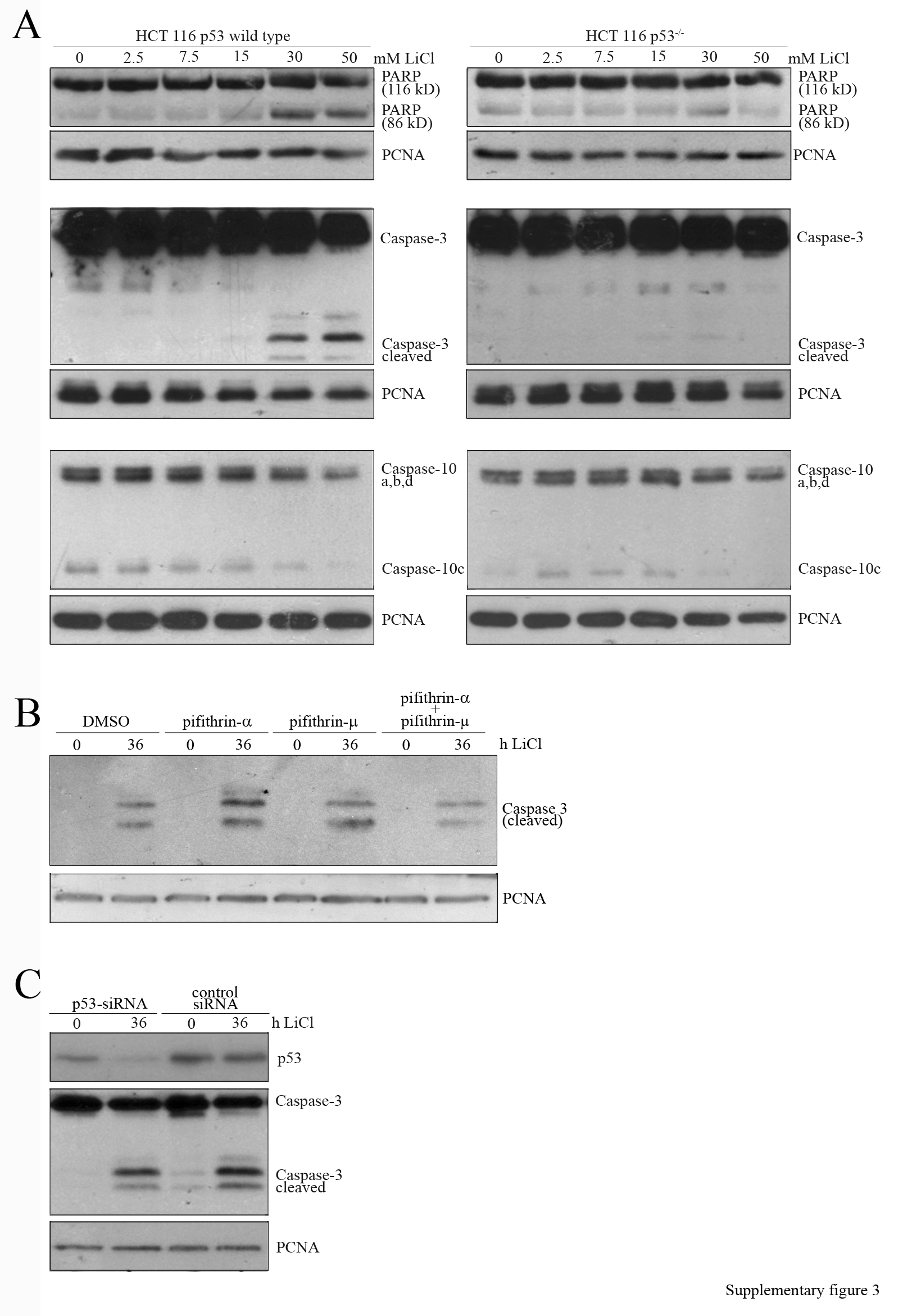

Supplement: Additional file 3 — Figure S3 - PARP and Caspases are cleaved after treatment with LiCl. (A) HCT116 wild type and HCT116 p53-/- cells were incubated with the indicated doses of LiCl for 48 hours. Cell lysates were prepared and 50 μg of protein were separated on a 10% SDS-PAGE gel. Proteins were transferred onto a PVDF membrane and probed with an antibody directed against PARP, Caspase-3 and Caspase-10, or against PCNA for a loading control. (B) HCT116 wild type cells were incubated with 50 μM pifithrin-α, 10 μM pifithrin-μ, with both, or with vehicle (DMSO) for control. After 4 hours, 50 mM LiCl were added. 36 hours after the addition of LiCl, cell lysates were prepared and 50 μg of protein were separated on a 15% SDS-PAGE gel. Proteins were transferred onto a PVDF membrane and probed with an antibody directed against Caspase-3, or against PCNA for loading control. (C) U2OS cells were transfected with siRNA targeted against p53 or with a control siRNA. 24 hours after transfection 50 mM LiCl were added. 36 hours after the addition of LiCl, cell lysates were prepared and 50 μg of protein were separated on a 15% SDS-PAGE gel. Proteins were transferred onto a PVDF membrane and probed with an antibody directed against Caspase-3, or against PCNA for a loading control. [file 1478-811X-9-15-S3.TIFF]

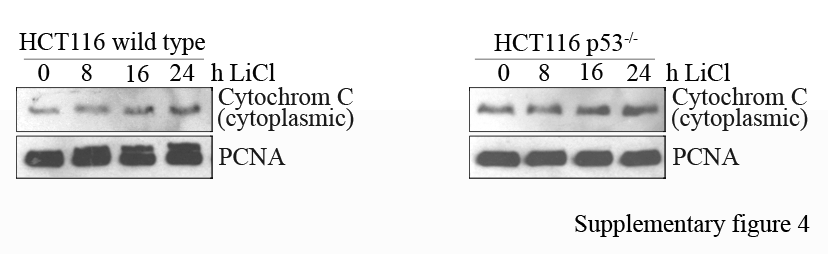

Supplement: Additional file 4 — Figure S4 - LiCl does not induce the release of Cytochrome C from mitochondria. HCT116 wild type and HCT116 p53-/- cells were incubated with 50 mM LiCl for the indicated time. Cells were lysed and mitochondria were separated from the cytoplasmic fraction by centrifugation. 50 μg of protein of the cytoplasmic fraction were separated on a 15% SDS-PAGE gel, transferred onto a PVDF membrane and probed with an antibody directed against cytochrome C, or PCNA for a loading control. [file 1478-811X-9-15-S4.TIFF]

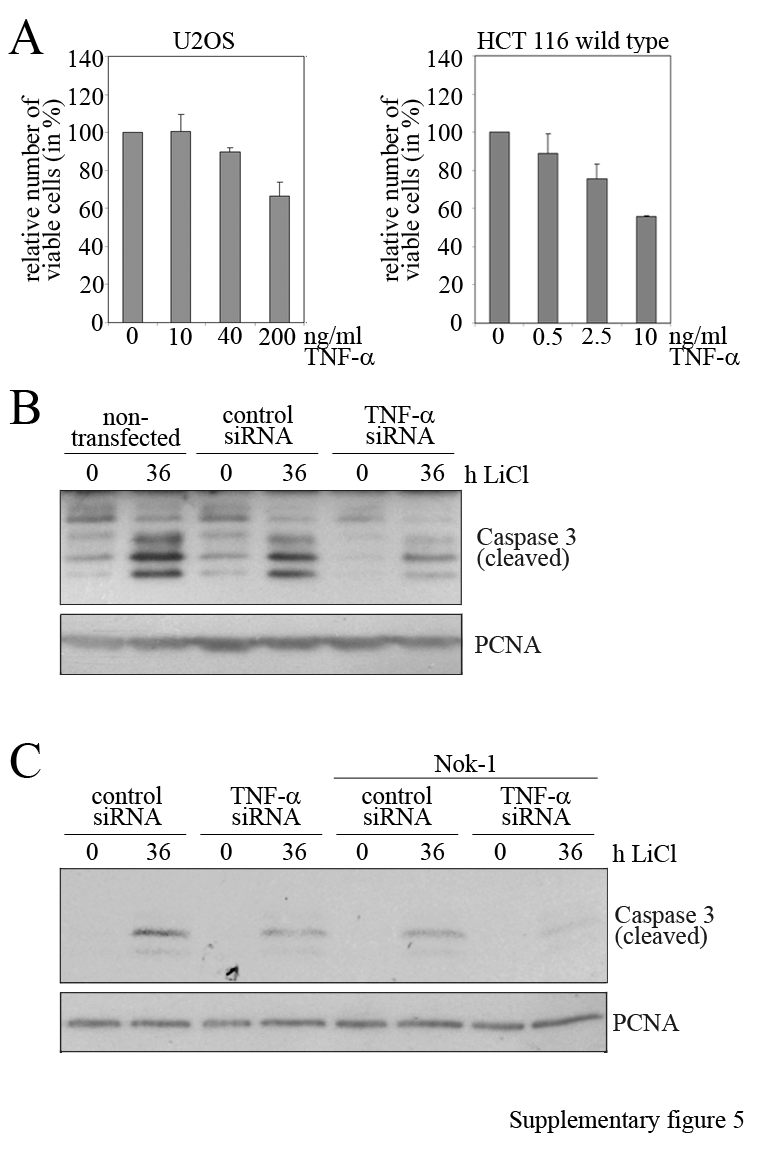

Supplement: Additional file 5 — Figure S5 - Caspase 3 cleavage and cell survival in dependence of TNF-α and FasL. (A) U2OS and HCT116 wild type cells were plated in 96-well plates at a density of 1 × 10^3^ cells per well. 24 hours after plating, TNF-α was added to the indicated concentrations. 72 hours after drug addition, the relative number of viable cells was determined by MTT-assay. Mean values and standard deviations of three independent experiments were calculated and plotted. The relative number of cells in the absence of TNF-α was set to 100%. (B) HCT116 wild type cells were transfected with siRNA targeted against TNF-α or with a control siRNA, or left untransfected for control. 24 hours after transfection, 25 mM LiCl were added and the cells were incubated for further 36 hours. Cells were lysed and 50 μg of protein were separated on a 15% SDS-PAGE gel. Proteins were transferred onto a PVDF membrane and probed with antibodies directed against cleaved Caspase-3, or PCNA for a control. (C) U2OS were transfected with siRNA targeted against TNF-α or with a control siRNA. 20 hours after transfection, Nok-1 antibody was added at a dilution of 1:500 where indicated. Four hours later, 50 mM LiCl were added and the cells were incubated for a further 36 hours. Cells were lysed and 50 μg of protein were separated on a 15% SDS-PAGE gel. Proteins were transferred onto a PVDF membrane and probed with antibodies directed against Caspase-3, or PCNA for a control. [file 1478-811X-9-15-S5.TIFF]

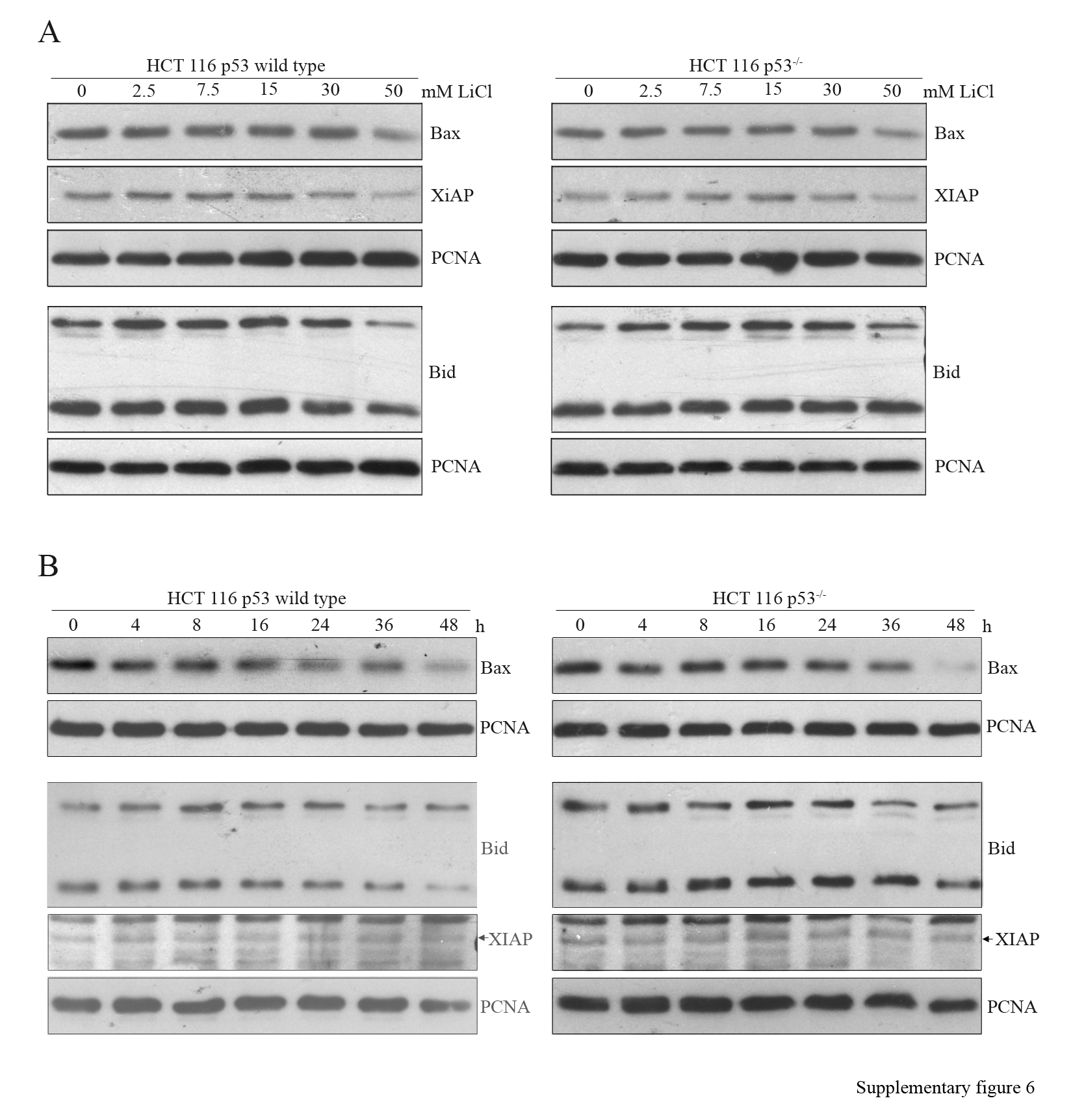

Supplement: Additional file 6 — Figure S6 - S6: Treatment with LiCl does not affect expression of Bax, XIAP or Bid. (A) HCT116 wild type and HCT116 p53-/- cells were incubated with the indicated doses of LiCl for 48 hours. Cell lysates were prepared and 50 μg of protein were separated on a 10% SDS-PAGE gel. Proteins were transferred onto a PVDF membrane and probed with antibodies directed against Bax, XIAP and Bid, or against PCNA for a loading control. (B) HCT116 wild type and HCT116 p53-/- cells were incubated with 50 mM LiCl for the indicated times. Cell lysates were prepared and 50 μg of protein were separated on a 10% SDS-PAGE gel. Proteins were transferred onto a PVDF membrane and probed with antibodies directed against Bax, XIAP and Bid, or against PCNA for a loading control. [file 1478-811X-9-15-S6.TIFF]
